# Built-in Timer Delays Differentiation

**DOI:** 10.1371/journal.pbio.1001254

**Published:** 2012-01-31

**Authors:** Liza Gross

**Affiliations:** Senior Science Writer/Editor, Public Library of Science, San Francisco, California, United States of America

## Abstract

In response to sudden environmental stress, <i>B. subtilis</i> cells can defer sporulation for multiple cell cycles using a pulsed positive feedback loop.

**Figure pbio-1001254-g001:**
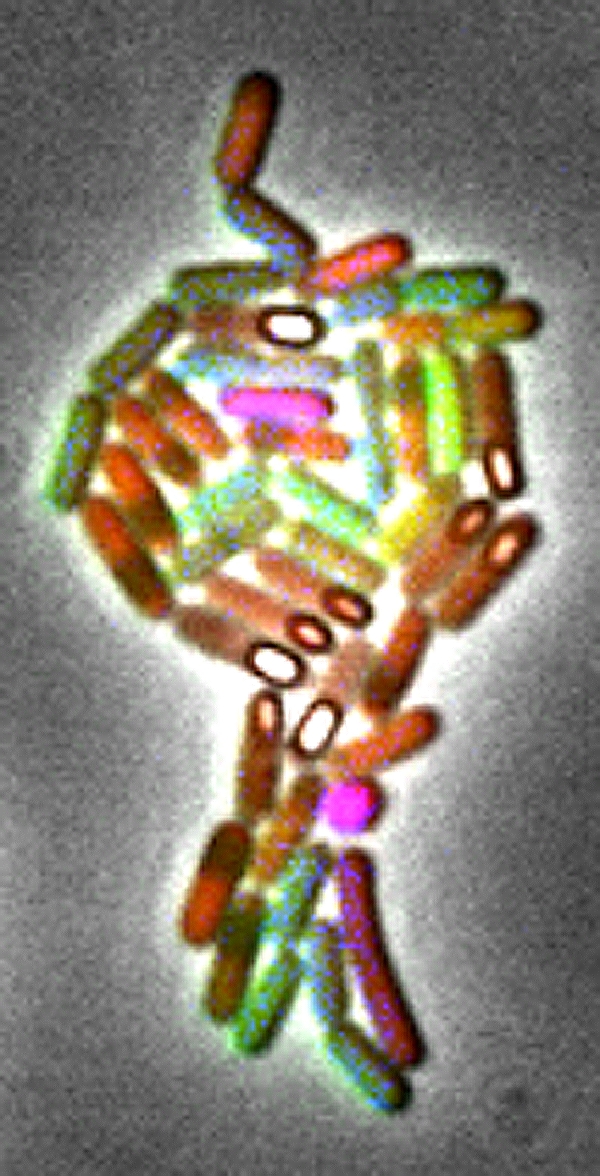
***Bacillus subtilis*** **can cope with stress by transforming into a dormant spore. A pulsed genetic timing circuit enables cells to defer transformation and undergo multiple rounds of proliferation before sporulating.**

[Fig pbio-1001254-g001]“Timing is everything” may be one of the most overused clichés in developmental biology, but not without reason. From the moment a fertilized egg starts to divide—initially at a breakneck pace, then dramatically slower as thousands of nascent cells sort into layers—cells follow an exacting schedule that tells them when to divide, differentiate into a specialized cell, and assemble into emerging tissues, organs, and structures. Ill-timed responses can result in malformed limbs or prove fatal if the misstep occurs during a critical developmental window.

Single-celled organisms like bacteria likewise must manage time-sensitive responses to life-threatening challenges in their environment. Some species of bacteria respond to stress, notably starvation, by forming spores, metabolically inert structures capable of surviving extreme conditions.

While some cells respond to environmental signals right away, others take much longer. Such deferred responses have been described in developing rodents and frogs, where cells postpone differentiation for several rounds of proliferation, and in bacterial colonies, where cells rely on signals from their neighbors before settling on a course of action. How cells manage these delayed responses remains a relatively unexplored question.

In a new study published this week in *PLoS Biology*, researchers led by Michael Elowitz worked with the spore-forming bacteria *Bacillus subtilis* to investigate the mechanisms that cells use to delay differentiation. When timing is everything, it turns out, it helps to have a reliable timer. Just as some grenades have a fuse-controlled timer that delays detonation for several seconds after removing the safety pin, *B. subtilis* bacteria, the team discovered, have internal timers that delay sporulation over several rounds of cell division after losing a food source.

Working with *B. subtilis* offers the advantage of working with a pared-down differentiation system—a single cell turns into a spore—where the genetic networks are well known. Stressful conditions trigger a signaling pathway that activates the “master regulator” SpoOA, a transcription factor that controls the expression of genes involved in sporulation, through a chemical modification known as phosphorylation. Sporulation takes place when concentrations of phosphorylated Spo0A reach a critical threshold that trips the sporulation gene circuitry. But in some cases, Spo0A concentrations build slowly, over several cell cycles, allowing cells to multiply before seeking refuge in dormancy.

Biologists have long been able to watch cells swirl, twirl, and tumble into rushing cellular rivulets as they differentiate and assemble into the emerging structures of a developing organism thanks to time-lapse video microscopy. Elowitz and his team used the same technology to observe the molecular machinations of individual bacterial cells differentiating into spores to avoid starvation.

The researchers found that individual cells went though five rounds of division before sporulating and that the cells achieve this delay by setting a timer. To find out how the timer works and what controls it, they tagged an Spo0A target gene with a fluorescent protein, allowing them to track the activity of the transcription factor over time. They saw “discrete pulses” of Spo0A activity, once per cell cycle, in cells denied nutrients. These pulses grew over each cell cycle until sporulation, suggesting the pulses act like a ticking time bomb in reverse: instead of counting down the minutes to detonation, each pulse cranks up the levels and activity of Spo0A, delaying sporulation until concentrations reach the critical threshold.

Faced with extreme conditions, the results suggest, bacteria configure an internal timer (which the authors describe as a pulsed positive feedback loop) that counts five cell cycles before triggering sporulation. The delay might buy cells a little time in case conditions change or the opportunity arises to try other survival strategies—such as forming a community with other cells as a biofilm or, conversely, joining forces with other cells to cannibalize their neighbors for sustenance—before resorting to more drastic measures. Delaying dormancy could also afford individual bacteria strains time to increase their numbers, giving them a competitive advantage over cells that push the sporulation panic button.

The secret to the timer's accuracy, the authors explain, lies in the finding that the timer mechanism operates as a feedback loop in discrete phases. While most genetic processes respond to stimuli within the span of a single cell cycle, the increasing bursts of Spo0A activity provide a way for the cell to extend its response over multiple cycles. And because different parts of the sporulation network are activated during each cycle, the timer could be less sensitive to interference from normal fluctuations within the cell.

Although the timer described in this study operates in bacteria, timers controlled by individual cells function during vertebrate development as well, allowing cells to delay decisions to adopt specific fates while they proliferate. It's unclear whether these intracellular timing mechanisms occur in other pathways or how they might work. But with so many biological processes where timing is critical—from development to senescence—evidence of novel strategies to buy time may show up in surprising places.

**Levine JH, Fontes ME, Dworkin J, Elowitz MB (2012) Pulsed Feedback Defers Cellular Differentiation. doi:10.1371/journal.pbio.1001252**

